# Cold Plasma-Induced Changes in Polyethylene Particles and Their Binding Affinity to Selected Pharmaceuticals

**DOI:** 10.3390/molecules30183756

**Published:** 2025-09-16

**Authors:** Aleksandra Wypart-Pawul, Beata Karwowska, Renata Caban, Anna Grobelak

**Affiliations:** 1 Faculty of Infrastructure and Environment, Częstochowa University of Technology, 42201 Częstochowa, Poland; a.wypart-pawul@pcz.pl (A.W.-P.); beata.karwowska@pcz.pl (B.K.); 2 Faculty of Production Engineering and Materials Technology, Częstochowa University of Technology, 42201 Częstochowa, Poland; renata.caban@pcz.pl

**Keywords:** cold plasma, polyethylene microplastics, pharmaceutical residues, sorption, environmental chemistry, advanced oxidation

## Abstract

Environmental contamination with microplastics and trace pharmaceuticals is an increasing ecological and health concern. This study aimed to investigate the effects of low-temperature cold plasma on polyethylene (PE) microplastic particles and to assess the potential for degradation of pharmaceuticals adsorbed onto their surfaces. Two types of PE samples were prepared: suspended in distilled water and in treated wastewater. All samples were exposed to cold plasma. In the second stage, PE particles were saturated with selected pharmaceuticals (diclofenac, sulfamethoxazole, trimethoprim) and then subjected to plasma treatment. Pharmaceutical concentrations were measured using high-performance liquid chromatography (HPLC). Particle morphology was analyzed via light microscopy (after Nile red staining) and scanning electron microscopy (SEM). The results showed that cold plasma treatment leads to agglomeration of PE particles, with the extent increasing with longer plasma exposure time. Pharmaceuticals adsorbed to the PE surface in the range of 20–70% of the applied dose. Cold plasma demonstrated the ability to remove pharmaceutical contaminants, particularly diclofenac (>98%), sulfamethoxazole (99.99%) and trimethoprim (>98%). These findings indicate that cold plasma has promising potential as a supportive technology for removing both microplastics and pharmaceutical residues from wastewater and aquatic environments.

## 1. Introduction

The presence of microplastics in wastewater and surface waters poses a growing threat to aquatic ecosystems and public health. Their ability to interact with pharmaceutical compounds can lead to the formation of persistent complexes that increase the mobility and toxicity of contaminants. Microplastic particles act as a potential vector, adsorbing various substances, chemical compounds and pathogens on their surface [1]. Microplastics in the aquatic and wastewater environment originate from two main sources: primary and secondary. Primary microplastics are intentionally manufactured small plastic particles used in products like cosmetics, detergents, and pharmaceuticals, typically in the form of microbeads or capsules with defined sizes. In contrast, secondary microplastics are formed through the degradation of larger plastic waste (e.g., bags, bottles, films, synthetic fibers) due to physical, chemical, or biological processes (like UV radiation, abrasion, or microbial activity), leading to particles that can interact with other pollutants, including pharmaceuticals. Microplastics ranging in size from 1 to 1000 µm are classified as conventional microplastics (MPs), representing an intermediate category between nanoplastics (<1 µm) and larger plastic debris (>1 mm). Scientific literature often distinguishes two subgroups within this range: 1–100 µm, considered very fine particles capable of penetrating biological tissues, and 100–1000 µm, which exhibit different sorption behaviors toward pollutants and are more easily detected under a microscope [2,3].

Plastics are characterized by exceptional durability, which under favorable environmental conditions may extend from hundreds to even thousands of years. Along with the dynamic increase in plastic waste, the scale of ecological threats is also intensifying. The widespread dispersal of microplastics is largely a consequence of human activity. Although the degradation of polymers may appear desirable from an environmental perspective, it does not automatically eliminate their harmfulness. Under the influence of atmospheric factors, microbial activity, and mechanical damage, plastic products gradually undergo structural and property changes, fragmenting into increasingly smaller particles. Contrary to expectations, this process is not beneficial, as micro- and nanoparticles readily spread throughout the environment, and, in parallel, various additives originally incorporated to impart specific properties to plastics are released during the breakdown of polymer networks. The presence of polyethylene microplastic particles in wastewater and surface waters has serious ecological and health consequences, especially in the context of the interaction of these particles with pharmaceuticals. There are a number of techniques and methods for detecting, identifying and quantifying microplastic particles [4]. The most commonly used methods include microscopic infrared spectroscopy (FTIR), Raman spectroscopy, and pyrolysis. Increasingly, multi-step strategies are being employed, combining initial visualization (optical microscopy) with subsequent spectroscopic analysis (micro-FTIR or Raman) and pyrolysis, which enhances both the quantitative and qualitative accuracy of microplastic identification [5]. Research is also being conducted on the impact of microplastic particles on living organisms such as earthworms [6].

Microplastics are an integral component of wastewater and should be taken into account when designing advanced, fourth-stage treatment systems. They constitute a contaminant in their own right, but also act as carriers for organic pollutants and microorganisms, including pathogens. One of the most promising methods for addressing such complex wastewater contamination is the application of cold plasma technology, which offers advanced capabilities for the degradation of persistent pollutants. This method is applied in food processing [7], surface cleaning [8], and sterilization [9], as well as in the removal of selected micropollutants from wastewater [10]. Using this technology allows for the controlled introduction of oxygen groups (C-O, C=O), roughening the surface, and lowering the molecular weight. This translates into susceptibility to further degradation and modifies sorption properties. Cold plasma, as a source of reactive oxygen and nitrogen species (ROS/RNS), demonstrates high application potential for surface modification of polymeric materials, including microplastics. Due to its low temperature and operation without the need for aggressive chemicals, this method is safe for the structure of the treated material while effectively inducing chemical changes such as increased polarity or the introduction of functional groups [11]. Applying cold plasma in studies on microplastics allows for a better understanding of how modified polyethylene surfaces affect their ability to sorb pollutants, including pharmaceutical compounds. The high controllability of the process and its environmentally friendly nature provide additional justification for choosing this technology in the context of the research problem. Despite growing research on the effects of cold plasma (CP) on microplastics and pharmaceuticals, several critical knowledge gaps remain. In particular, the long-term stability of plasma-modified microplastics in environmental conditions is still poorly understood. The potential formation and toxicity of degradation byproducts resulting from CP treatment have not been fully assessed. Additionally, the scalability of CP processes for practical wastewater treatment applications remains largely unexplored. Addressing these gaps is essential to evaluate the environmental relevance and practical feasibility of plasma-based remediation strategies.

The aim of the presented research was to evaluate the effectiveness of cold plasma treatment as a promising approach for the removal of pharmaceuticals and microplastics from wastewater. In particular, the research focused on the sorption behavior of selected pharmaceuticals on polyethylene particles and how this behavior was altered after low-temperature plasma treatment. By addressing both degradation processes and adsorption phenomena, the study provides a relevant and timely contribution to understanding the potential of plasma-based technologies in mitigating emerging environmental contaminants.

## 2. Results

**Figure 1:**
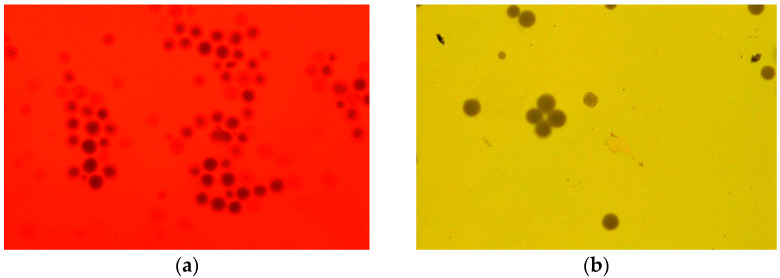
Polyethylene particles stained with Nile Red under UV light; observed against a red background using a red barrier filter, applied for the detection of red-range fluorescence and elimination of shorter-wavelength excitation light (**a**), and observed against a yellow background using a UV barrier filter (**b**). The polyethylene particles appear as spherical, black spheres opaque to light.

As a result of the conducted studies, a dataset was obtained that enables the assessment of the effects of plasma treatment on the properties of polyethylene particles in different matrices. Both morphological features and changes in particle behavior as a function of cold plasma exposure time were analyzed. Based on basic observations under an optical microscope, both in water-based samples and treated wastewater, it was found that polyethylene particles do not stain with Nile Red. In the field of view, they appear as black, light-impermeable particles (Figure 1). This is most likely due to their high density as a reference material. Polyethylene (PE), especially high-molecular-weight, smooth, and crystalline forms, has low surface energy and a very limited capacity to sorb dyes.

### 2.1. Microscopic Analysis of Polyethylene Particles in Water

**Figure 2:**
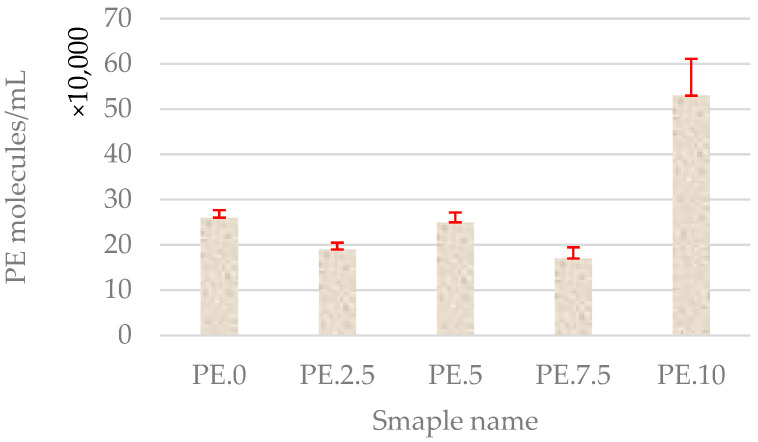
Number of PE particles per 1 mL of sample based on hemocytometer chamber calculations in water samples with marked standard deviation. The observed increase in the number of objects after plasma treatment indicates fragmentation of larger PE particles into smaller agglomerates, which may indicate the effect of cold plasma on particle morphology; number of replications (N = 3).

Through the analysis of water samples containing a polyethylene standard in a glass hemocytometer counting chamber, the number of PE particles per milliliter of sample was determined. Observations indicated that, after the longest exposure time of the sample to the plasma stream, the number of visible particles in the field of view increased (Figure 2).

**Figure 3:**
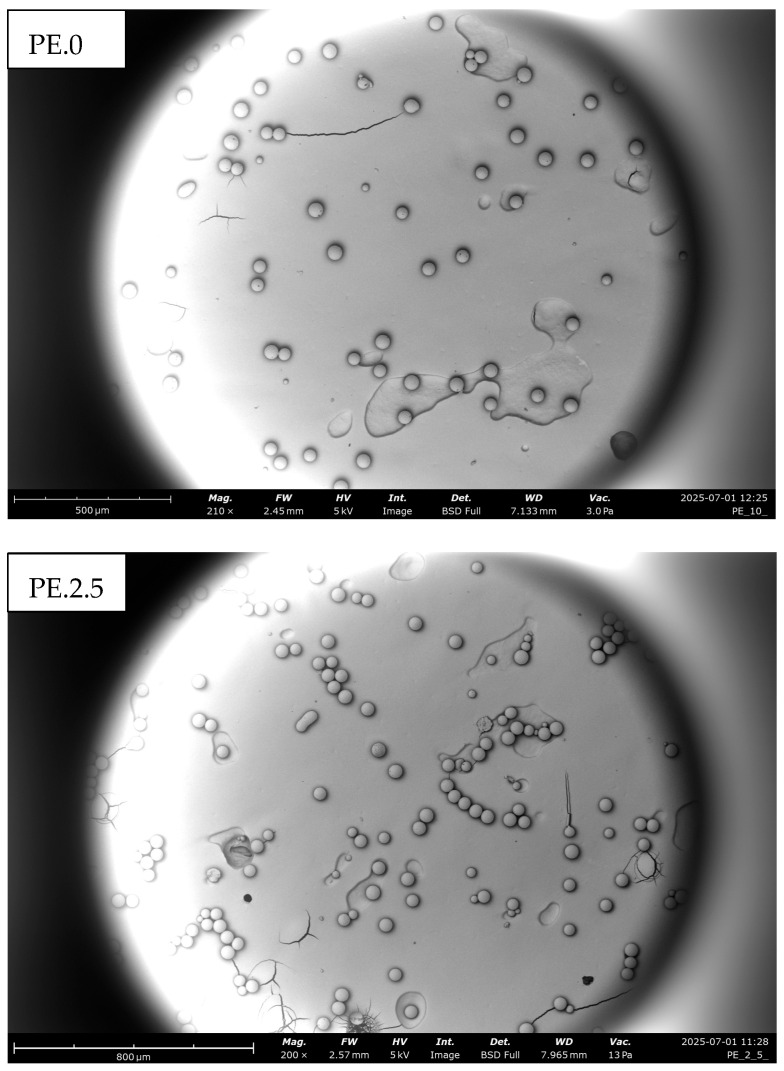

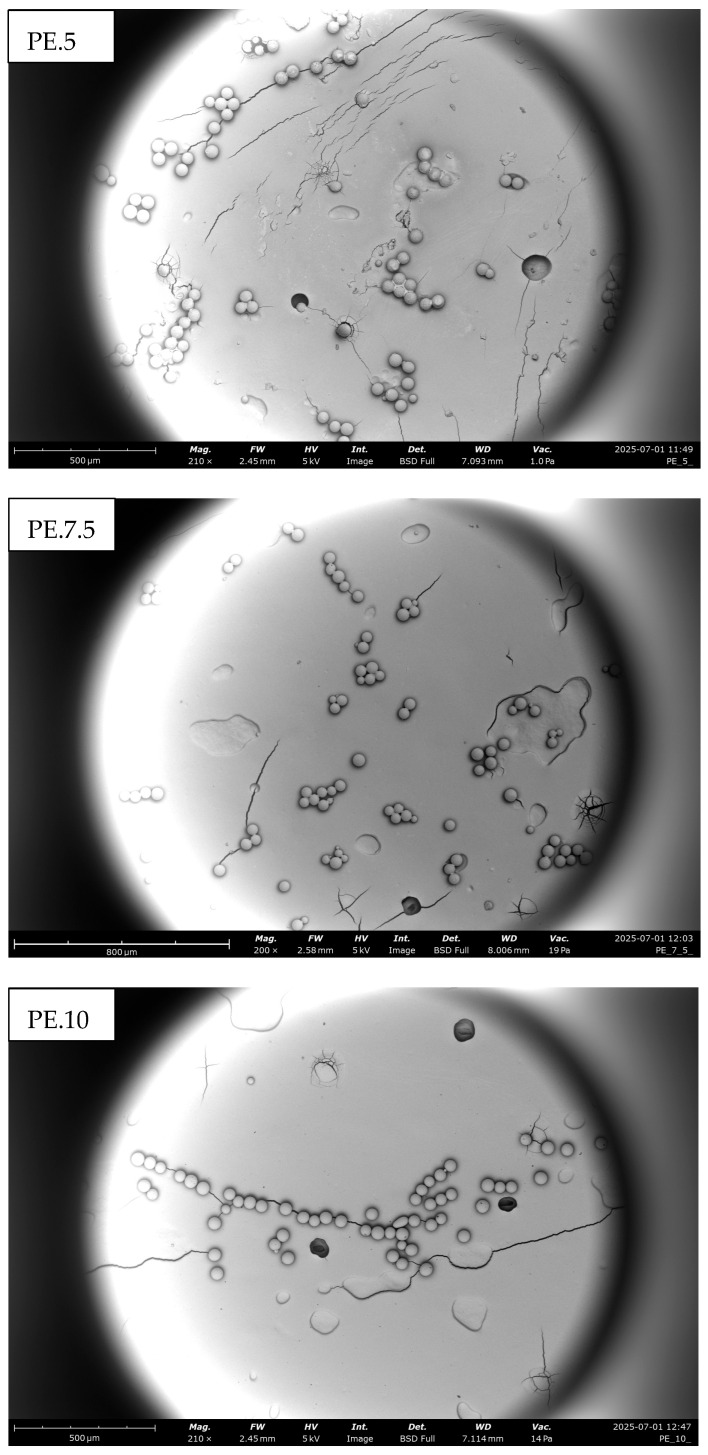
Agglomerations of PE particles formed as a result of CP action over time observed under SEM. The progressive surface modification and clustering illustrate how plasma exposure alters particle morphology and promotes the formation of secondary microplastic structures relevant to environmental behavior.

This was due to the formation of increasingly larger clusters, as the particles aggregated into progressively larger agglomerates. This tendency was also confirmed by SEM images (Figure 3). As a result of prolonged plasma treatment, individual microplastic particles began to cluster together, forming larger and more compact aggregates. This suggests that cold plasma may alter the surface properties of polyethylene, enhancing intermolecular interactions and potentially facilitating their removal from aquatic environments.

**Figure 4:**
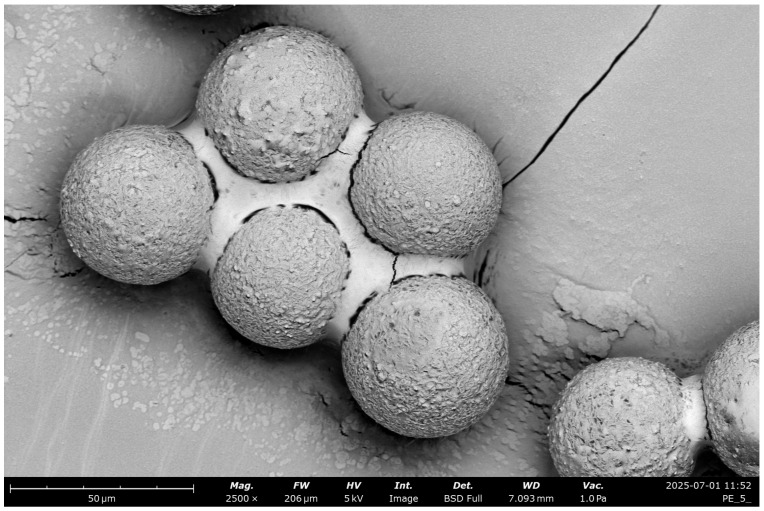
Scanning electron microscopy (SEM) visualization of PE particles aggregated into compact clusters following prolonged exposure to cold plasma in an aqueous environment. The formation of dense agglomerates indicates structural reorganization of the polymer surface, demonstrating how extended plasma treatment can drive secondary aggregation processes with potential implications for microplastic stability and transport in aquatic systems.

The application of cold plasma in a distilled water environment caused a noticeable change in the behavior of polyethylene standard particles. As shown in the SEM image (Figure 4), initially dispersed spherical PE particles began to form larger and more compact agglomerates following exposure to the plasma stream. This process intensified with increasing CP exposure time. The high-resolution SEM image reveals tightly packed spherical particles that underwent aggregation, showing a clear reduction in interparticle distances. This change indicates an increase in adhesion forces between particles. Furthermore, surface dulling and increased roughness were also observed, which may result from partial oxidation or the action of reactive plasma species (such as ROS or RNS), leading to modifications in the surface topography of PE. A probable mechanism behind agglomerate formation is the emergence of functional groups (e.g., hydroxyl, carbonyl) on the surface of PE particles, which increases their hydrophilicity and surface energy. As a result, intermolecular interactions such as van der Waals forces or potential hydrogen bonding are intensified, leading to their self-organization into larger structures.

**Figure 5:**
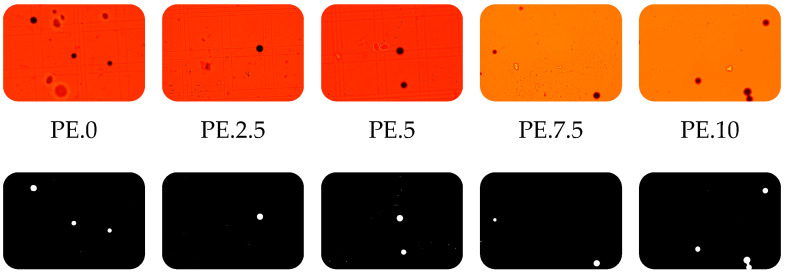
Visualization of the measured polyethylene molecules in a binary image in the Fiji program (ImageJ 1.54p) for quantitative analysis. This image processing step enables detection and measurement of particle clusters, providing the basis for assessing the impact of cold plasma treatment on particle number and size distribution.

The observed aggregation phenomenon may have significant environmental implications, particularly in the context of microplastic removal technologies. Larger and heavier agglomerates may be more easily separated using sedimentation, filtration, or flotation processes. As part of the morphological analysis, measurements of selected polyethylene particles were conducted using Fiji software (ImageJ 1.54p). During microscopic observations, fields of view with the smallest possible number of PE particles were selected to facilitate further image processing and accurate determination of particle sizes. The analysis showed that the diameter of individual particles prior to aggregation ranged from 10 to 45 µm (Table 1). After CP treatment, no significant changes in the size of individual particles were observed, indicating that plasma exposure does not cause their shrinkage or elongation (Figure 5). These findings confirm that the effect of cold plasma is mainly focused on the surface modification of PE particles and their tendency to form larger structures through aggregation.

**Figure 6:**
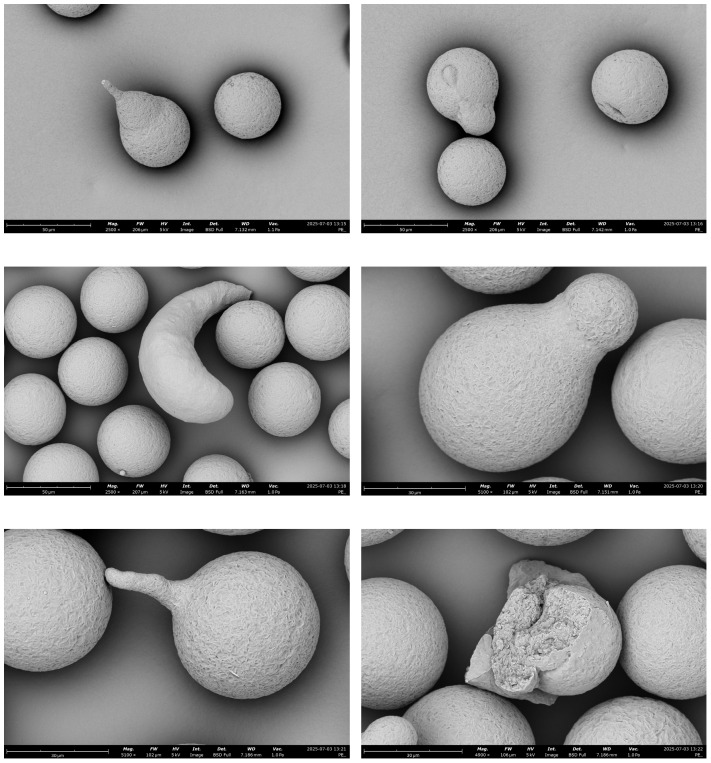
Surface morphology of dry polyethylene (PE) standard without cold plasma (CP) treatment. The limited particle deformations observed here serve as a control reference, highlighting the baseline structural features of PE prior to plasma exposure and enabling comparison with the pronounced modifications induced by CP treatment.

During the analysis of SEM images, the presence of PE particles with irregular, deformed shapes, deviating from the typical spherical morphology of the standard, was also observed. Such particles were visible in nearly every analyzed sample, regardless of the cold plasma exposure time. To ensure that these deformations were not caused by CP treatment, control observations were carried out on dry PE standard samples that had not undergone any plasma treatment. Irregularly shaped particles were also identified in these control samples, clearly indicating that cold plasma is not responsible for the degradation or structural breakdown of PE particles (Figure 6). This suggests that the observed deformations are likely of primary origin, resulting from manufacturing inconsistencies in the standard or prior physicochemical processes.

**Figure 7:**
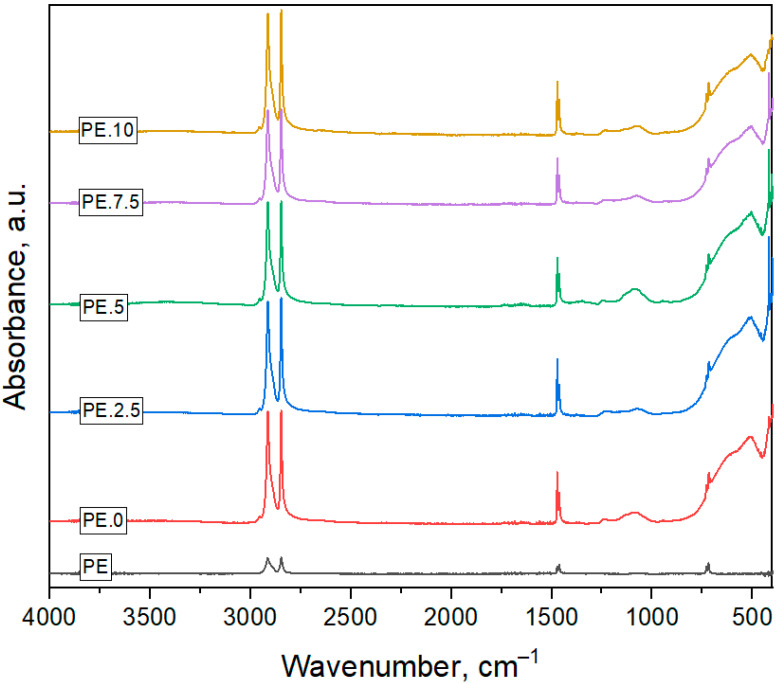
FTIR-ATR spectra of polyethylene (PE) particles before (PE.0) and after different durations of cold plasma treatment (PE.2.5, PE.5, PE.7.5, PE.10). All spectra show characteristic absorption bands of PE. After exposure to plasma, additional bands appear in the regions 1700 cm^−1^ and 1000–1200 cm^−1^, which can be assigned to oxygen-containing functional groups.

The strongest peaks (observed in all tested samples) at wavenumbers 2918 cm^−1^ and 2849 cm^−1^ are attributed to the asymmetric and symmetric stretching vibrations of methylene groups (–CH_2_–) in polyethylene (PE) (Figure 7). Peaks observed at 1471 cm^−1^ and 1462 cm^−1^ correspond to bending vibrations of methylene groups. Additional characteristic polyethylene bands appear at 729 cm^−1^ and 719 cm^−1^, which are assigned to the rocking vibrations of PE methylene groups [12].

In the case of samples exposed to plasma treatment and pharmaceuticals, the appearance of new bands in the range of 1700–1000 cm^−1^ and 600–500 cm^−1^ is observed [13,14,15]. This indicates the formation of new chemical bonds or the presence of adsorbed molecules, resulting from interfacial interactions between the polyethylene and the pharmaceutical compounds.

### 2.2. Microscopic Analysis of Polyethylene Particles in Treated Wastewater

**Figure 8:**
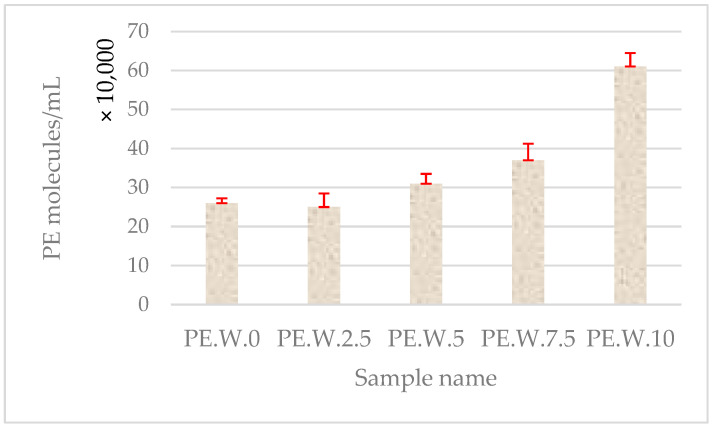
Number of PE particles per 1 mL of sample based on hemocytometer chamber calculations in treated wastewater with marked standard deviation. The observed increase in the number of objects after plasma treatment indicates fragmentation of larger PE particles into smaller agglomerates, which may indicate the effect of cold plasma on particle morphology; number of replications (N = 3).

Microscopic observations of samples containing treated wastewater with polyethylene in a hemocytometer counting chamber revealed an increasing trend in the number of particles observed within the field of view (Figure 8). This was associated with the formation of PE aggregates induced by cold plasma treatment. The number of visible particles increased with prolonged exposure of the sample to the CP stream.

It should be emphasized that the particle counts presented in Figure 2 and Figure 8 refer to clusters rather than individual polyethylene particles. The observed increase in the number of objects after plasma treatment most likely reflects fragmentation of larger pieces into smaller agglomerates, which are subsequently recognized as separate clusters. While this approach provides qualitative insight into fragmentation phenomena, it does not fully capture particle aggregation dynamics. Determination of size distribution parameters would allow for a more quantitative assessment, and such analyses are planned for future studies.

**Figure 9:**
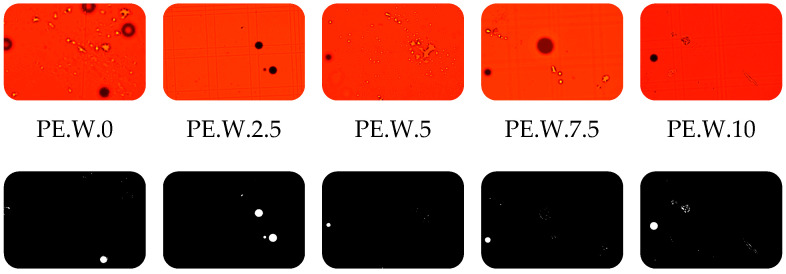
Visualization of measured polyethylene molecules in binary image in Fiji program (ImageJ 1.54p) in PE.W samples. This image analysis approach allows for consistent identification of particle clusters and facilitates quantitative comparison, thereby supporting the evaluation of cold plasma effects on microplastic behavior.

As in the water-based samples, selected polyethylene particles were measured using Fiji software. During microscopic observations, fields of view containing the lowest possible number of PE particles were selected. The analysis showed that the diameter of individual particles prior to aggregation ranged from 10 to 45 µm (Table 2). After CP treatment, as in the water-based samples, no significant changes in the size of individual particles were observed, indicating that plasma exposure does not alter the shape of the particles (Figure 9). This confirms that the effect of CP is mainly focused on surface modification of PE and influences the tendency of particles to form larger structures.

All obtained results indicate that cold plasma does not cause degradation or deformation of polyethylene particles. Its effect is limited to surface modification, which leads to the intensification of aggregation processes. The observed phenomena suggest that CP may serve as an effective tool for supporting microplastic removal processes by increasing the tendency of particles to cluster into larger, more easily separable structures.

### 2.3. Pharmaceutical Binding to Polyethylene Particles Pre- and Post-Cold Plasma Treatment

**Figure 10:**
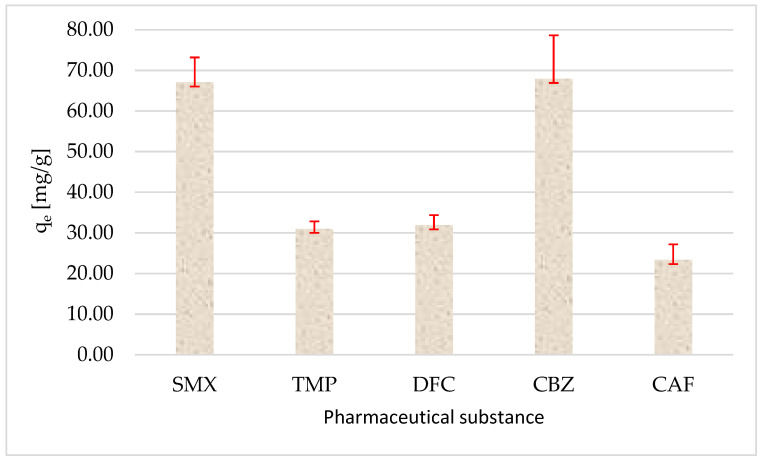
The amount of pharmaceutical adsorbed (q_e_) on PE particles after 24 h of sorption with marked standard deviation. The data illustrate how surface properties of PE influence sorption capacity, providing insight into the effect of cold plasma treatment on the interaction between pharmaceuticals and microplastic particles; number of replications (N = 3).

Initial experiments assessed the sorption behavior of selected pharmaceutical compounds: diclofenac (DCF), sulfamethoxazole (SMX), trimethoprim (TMP), carbamazepine (CBZ), and caffeine (CAF), on polyethylene particles in distilled water. After 24 h of shaking, sorption efficiencies varied considerably among the compounds, reflecting differences in their physicochemical properties (e.g., hydrophobicity, molecular structure, and functional groups). Each drug was tested at a nominal initial concentration of 20 mg/L, corresponding to the actual measured initial concentrations: 19.61 µg/mL (SMX), 19.07 µg/mL (TMP), 21.76 µg/mL (DFC), 24.35 µg/mL (CBZ), and 21.35 µg/mL (CAF). After 24 h of contact, the following equilibrium concentrations (Ce) were recorded: SMX 6.20 µg/mL, TMP 12.87 µg/mL, DFC 15.38 µg/mL, CBZ 10.76 µg/mL, and CAF 16.68 µg/mL. Based on these values, the amount of drug adsorbed per gram of PE (qe) was calculated (Figure 10).

**Figure 11:**
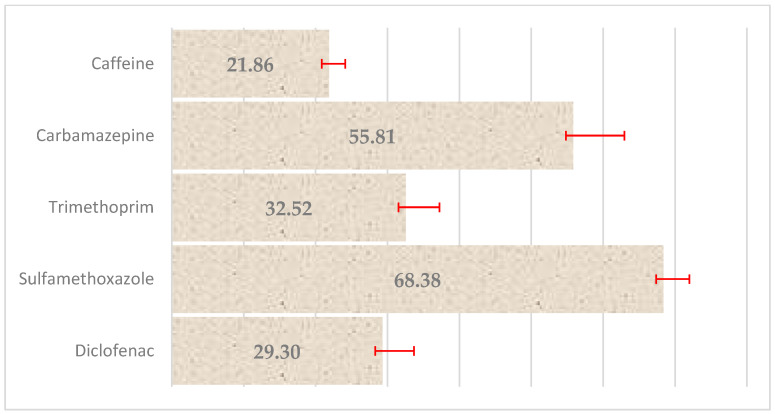
Percentage loss of selected pharmaceuticals from aqueous solution as a result of sorption on polyethylene with marked standard deviation. The results demonstrate the capacity of PE particles to remove pharmaceuticals from solution, highlighting the potential influence of surface modifications on adsorption efficiency and environmental behavior of microplastics; number of replications (N = 3).

This analysis confirmed that all five compounds exhibited measurable sorption onto the PE surface under the tested conditions. Among them, SMX and CBZ demonstrated the highest sorption efficiencies, while CAF and DFC showed lower removal rates. These findings suggest that even untreated polyethylene exhibits non-negligible sorptive capacity for certain pharmaceuticals, which may be further enhanced or altered by plasma-induced surface modification. The percentage loss of pharmaceuticals from the solution due to sorption on PE is shown in Figure 11.

**Figure 12:**
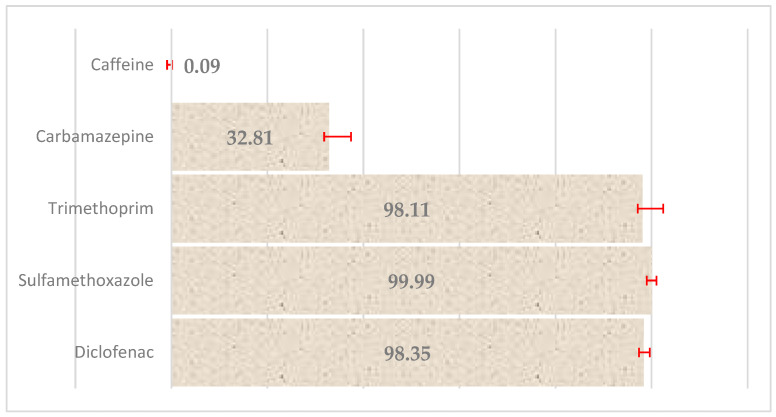
Percentage efficiency of reducing the concentration of selected pharmaceuticals under the influence of cold plasma with marked standard deviation. The data indicate the effectiveness of cold plasma in degrading or removing pharmaceuticals from aqueous solutions, demonstrating its potential application for wastewater treatment and mitigation of pharmaceutical contamination in the environment; number of replications (N = 3).

These results indicate that SMX and CBZ exhibited the highest affinity for PE, which is likely related to their moderate hydrophobic character and potential for π–π interactions with the polymer surface, i.e., the attraction between the aromatic systems of these pharmaceuticals and the polyethylene surface. In contrast, caffeine showed the lowest sorption efficiency, possibly due to its relatively higher polarity and lower. Subsequent experiments focused on the effect of cold plasma treatment on the degradation or further removal of the pharmaceuticals from the aqueous solutions containing PE particles. The most pronounced reductions in pharmaceutical concentrations were observed for SMX, DCF, and TMP, indicating that CP treatment significantly enhances the removal or transformation of these compounds. After 5 min of plasma treatment, the reductions in parent compound concentrations reached (Figure 12).

These findings suggest that cold plasma induces effective oxidative degradation of several pharmaceuticals, particularly those more susceptible to radical-mediated processes (e.g., SMX and DCF). The lower degradation efficiency for CBZ may be attributed to its known resistance to oxidative breakdown. Interestingly, caffeine exhibited an apparent increase in concentration post-treatment. Given that HPLC analysis was based on UV-absorbance without mass-selective detection, it is plausible that the observed peak includes caffeine metabolites or transformation products generated during plasma exposure, which co-elute under the applied chromatographic conditions. It should be emphasized that the pharmaceuticals were tested at a concentration of 20 mg/L, significantly higher than typical levels found in wastewater. This concentration was chosen to facilitate detection and reliably assess the underlying sorption and degradation mechanisms. Although the obtained removal efficiency values do not directly reflect the situation at environmental concentrations, the obtained mechanistic conclusions remain relevant and provide valuable guidance for future research and potential practical applications.

### 2.4. Energy Balance Analysis

The results obtained from the energy balance indicate the potential of cold plasma technology as an effective method for the removal of selected pharmaceutical micropollutants from water (Table 3). The use of a cold plasma generator demonstrates high energy efficiency in the degradation of pharmaceuticals such as sulfamethoxazole, diclofenac, and trimethoprim. In contrast, carbamazepine requires significantly higher energy input to achieve 1% reduction, indicating lower process efficiency for this compound. Caffeine did not undergo degradation under the tested conditions, suggesting the need to adjust process parameters for its effective removal. The SEC calculations and unit cost estimates enable the comparison of energy efficiency across different contaminants and support planning of treatment costs at an industrial scale.

The calculated energy cost for the analyzed samples with a volume of 50 mL was 0.03699 EUR. When normalized to 1% reduction in pharmaceutical concentration in the same volume, the energy cost was estimated at 0.00037 EUR for sulfamethoxazole (SMX), 0.00038 EUR for trimethoprim (TMP), 0.00038 EUR for diclofenac (DFC), and 0.00113 EUR for carbamazepine (CBZ). These values highlight the relatively low unit energy expenses associated with the removal of SMX, TMP, and DFC, whereas CBZ requires considerably higher costs, reflecting its lower susceptibility to cold plasma treatment.

## 3. Discussion

The application of cold plasma (CP) in both distilled water and treated wastewater resulted in comparable physicochemical responses of polyethylene (PE) particles, primarily manifested through aggregation phenomena and surface modification. Microscopic analyses revealed that, regardless of the aqueous matrix, prolonged plasma exposure consistently induced clustering of initially dispersed PE particles into more compact agglomerates. These changes are likely driven by the formation of polar functional groups (e.g., hydroxyl, carbonyl) on the particle surface, enhancing interparticle adhesion, an effect reported in earlier studies on plasma-treated polymers [16,17]. Interestingly, despite the significantly more complex chemical background of treated wastewater, including organic matter, ions, and potential trace contaminants, the aggregation trend remained stable and reproducible. This suggests that the cold plasma-induced modifications of the PE surface are robust and not significantly hindered by matrix composition. Such consistency is encouraging in the context of real-world applications, where environmental variability is a key challenge for emerging water treatment technologies [18]. Further support for the selective surface activity of CP comes from size analysis conducted with Fiji software (ImageJ 1.54p). In both environments, the diameter of individual PE particles remained within the pre-treatment range (10–45 µm), confirming the absence of structural degradation, melting, or mechanical fragmentation during plasma exposure. This aligns with existing reports that atmospheric pressure cold plasma primarily induces surface oxidation or etching, rather than bulk transformation [19,20]. The conducted studies indicate a surface-specific mechanism of action of CP in modifying polymeric microplastics. The continuous formation of larger agglomerates in matrices can increase sedimentation or filtration efficiency, making CP an effective pretreatment strategy in microplastic remediation processes.

The observed sorption capacity, highest for sulfamethoxazole and carbamazepine, moderate for trimethoprim and diclofenac, and lowest for caffeine, aligns well with findings reported in the recent literature concerning interactions between pharmaceuticals and microplastic particles. Xu et al. [21] showed that the sorption of SMX onto PE is dependent on pH and ionic strength, and equilibrium can be reached within several hours, with distribution coefficients in the range of 20–30 L/kg. This suggests that the 24 h contact time used in our study may have promoted efficient SMX sorption. Rovanajatovo et al. [22] also found that SMX sorption onto PE was partially irreversible, indicating strong interactions with the polymer surface. The high sorption of CBZ (over 55%) in our study can be attributed to its neutral form at near-neutral pH and the presence of aromatic moieties, which promote hydrophobic and π–π interactions with PE surfaces. The ionization state of the compound, solution pH, and the surface properties of the adsorbent strongly influence the extent of sorption [23]. In our case, differences in sorption efficiency can largely be attributed to these parameters. SMX, as an anionic molecule, may form favorable electrostatic interactions with positively charged sites on the PE surface, whereas caffeine, being more polar, exhibits lower affinity for the hydrophobic polymer matrix. Sorption mechanisms observed in the literature include both hydrophobic and electrostatic interactions. Studies [23] emphasize that pharmaceutical ionization, ionic strength, and solution pH are critical factors influencing sorption to micropolymers. In our study, differences between the compounds (e.g., SMX anionic at neutral pH vs. CBZ neutral) account for the observed sorption values. DCF, although in its ionic form, can partially sorb under certain conditions due to the presence of aromatic groups and hydrophobic segments. The characteristics of the obtained results suggest that the dominant sorption mechanism is physisorption, and the most appropriate model to describe it would likely be the Freundlich isotherm, which accounts for surface heterogeneity and nonlinear sorption behavior. To confirm this assumption, further sorption studies across a wider concentration range and mathematical fitting of isotherm models are recommended.

The high efficiency of cold plasma in reducing the concentration of SMX (nearly 100%), DCF (>98%) and TMP (>98%) confirms the power of this process in drug degradation [10,24]. Our previous investigations using cold plasma alone (without polyethylene particles) demonstrated significant degradation of pharmaceuticals in aqueous solutions, achieving 73% removal of sulfamethoxazole, 98% of trimethoprim, and 91% of diclofenac, whereas carbamazepine and caffeine exhibited removal efficiencies below 1%. These findings underscore that cold plasma treatment by itself contributes substantially to pharmaceutical elimination and justify the incorporation of plasma-only control tests in the current study to better deconvolute the contributions of adsorption versus oxidative degradation [10]. Furthermore, the recent review by Guo et al. [25] elucidates direct cold plasma and plasma-activated water oxidation mechanisms, thus providing theoretical backing for the mechanistic interpretation and experimental design improvements. The study by Rayaroth et al. [26] showed that both diclofenac and carbamazepine were degraded by CP, although CBZ showed significant oxidative stability. Our results (approximately 32.8% CBZ degradation after 10 min) confirm the known resistance of CBZ, as it is often administered as one of the most persistent drugs. The lower degradation efficiency of CBZ (~32%) reflects its known resistance to oxidative conditions, even in advanced AOP or plasma processes. Literature indicates that CBZ requires longer exposure and more intense conditions (e.g., catalysts) to achieve > 90% degradation [27]. The observed apparent increase in caffeine concentration after plasma treatment seems to indicate the formation of transformation products that co-elute with caffeine on the HPLC column. This phenomenon has been repeatedly reported in the context of advanced oxidation processes, where transformation leads to multiple intermediates that are difficult to separate with a UV detector [28]. Therefore, this suggests the need for LC-MS/MS analyses in future studies. Combining pharmaceutical sorption onto PE and its degradation using cold plasma may exhibit a synergistic effect: sorption onto polymer surfaces can localize contaminants, increasing their exposure to reactive oxygen species generated by CP. In our case, such synergy was observed for SMX, DCF, and TMP. However, in the case of CBZ, optimization of purification parameters (e.g., duration, power, or catalyst addition) may be necessary to improve degradation efficiency.

The observed phenomena suggest that cold plasma (CP) may serve as an effective tool for enhancing microplastic removal processes by promoting the aggregation of particles into larger, more easily separable structures. The application of CP in aqueous environments- both in distilled water and treated wastewater- proved to be efficient and reproducible, regardless of matrix complexity. Surface modifications induced by plasma treatment may potentially enhance not only the aggregation behavior but also the reactivity of polyethylene (PE) particles toward other contaminants, such as pharmaceutical compounds or heavy metals.

The obtained energy cost values confirm that cold plasma treatment can be considered an energy-efficient technology for the removal of selected pharmaceuticals. The unit costs calculated for SMX, TMP, and DFC were at a similarly low level, indicating that these compounds are susceptible to plasma-induced degradation at relatively low energy demand. In contrast, the markedly higher cost calculated for CBZ reflects its chemical stability and resistance to oxidation, which is consistent with previous studies reporting poor degradability of carbamazepine under advanced oxidation processes. These findings suggest that while cold plasma is highly promising for a broad spectrum of pharmaceuticals, its application to more recalcitrant compounds may require optimization of operating parameters or combination with complementary treatment methods. Furthermore, the cost analysis, although performed on a laboratory scale, provides a valuable reference point for assessing the feasibility of scaling up plasma-based water treatment technologies.

In the context of future research, it is advisable to further investigate the physicochemical mechanisms responsible for the initiation and stabilization of agglomerates and to conduct trials in natural aquatic environments (e.g., surface waters), where the presence of biofilms, ions, and natural organic matter may influence the effect of CP. Studies focusing on the durability of surface modifications and the possibility of selectively tuning plasma parameters to enhance the sorption efficiency for specific classes of pollutants could be of particular interest. The integration of CP with other water treatment technologies, such as membrane filtration or adsorption, may open new directions in environmental engineering and water purification strategies. The presented studies did not identify specific reactive species formed during cold plasma treatment. Reactive species (e.g., hydroxyl radicals, ozone, hydrogen peroxide, singlet oxygen) play a key role in contaminant degradation. Further studies using advanced detection techniques such as ESR spin trapping or probe assays will be necessary to obtain a more detailed mechanistic understanding of drug degradation pathways.

## 4. Materials and Methods

In the conducted study, two types of samples were used. The samples were prepared using distilled water and treated wastewater originating from a municipal wastewater treatment plant operating with a conventional activated sludge system. The 50 mL samples were enriched with a microplastic standard, spherical, white polyethylene (PE) beads purchased from Cospheric LLC (Somis, CA, USA), with a density of 1.25 g/cc and a size range of 10–45 µm.

To generate a low-temperature plasma stream, a Plasma TEC-X (PLX) generator from Tantec A/S (Lunderskov, Denmark) with a power of 550 VA and a single plasma nozzle was used. The discharge flame width of the PLX nozzle ranged from 8 to 14 mm. The mains voltage and frequency for the PLX generator ranged from 100 to 250 V and 50/60 Hz. Maximum air humidity was 80%. Atmospheric air was introduced as the discharge gas at a pressure of 4–8 bar. The plasma stream was applied above the liquid surface at a distance of 0.05 m from the liquid’s surface. During the generator’s operation, an exhaust system was used to remove the resulting gases.

Microscopic observations of the analyzed samples were carried out using two types of microscopes: an Olympus optical microscope (Olympus Europa SE & Co. KG, Hamburg, Germany) equipped with a UV lamp (ultraviolet light) and/or a scanning electron microscope (SEM) Phenom ProX, Thermo Fisher Scientific (Waltham, MA, USA). The study utilized the lipophilic organic dye Nile Red (Saint Louis, MO, USA), which exhibits fluorescence, with the aim of intercalating into the structure of PE particles during observation under an optical microscope. Samples after CP treatment were also observed using a Bürker Thoma chamber (Heinz Herenz Medizinalbedarf GmbH, Hamburg, Germany) with a volume of 0.0025 mm^2^.

In order to identify the characteristic functional groups present in the chemical structure of the tested materials, Fourier-transform infrared spectroscopy with attenuated total reflectance (FTIR-ATR) (SHIMADZU Irraffinity-1s, Kyoto, Japan) was used. The analysis of the FTIR spectrum provides information on vibrating molecules and their bonds with the immediate chemical environment. Spectral data were collected using a SHIMADZU spectrophotometer equipped with an ATR attachment featuring a diamond crystal. FTIR-ATR measurements were performed in the spectral range of 400 to 4000 cm^−1^. A total of 60 scans per measurement were recorded, with a spectral resolution of 1 cm^−1^.

For the studies related to the sorption of pharmaceuticals on PE particles, selected pharmaceuticals presented in Table 4 were used. The analysis of pharmaceutical concentrations was performed using HPLC (High-Performance Liquid Chromatography). Chromatographic analysis was carried out using the Vanquish™ Analytical Purification LC system from Thermo Fisher Scientific (Waltham, MA, USA), equipped with an integrated Vanquish fraction collector. The chromatographic column used was an Accucore C18 column (150 mm × 3 mm) with a particle size of 2.6 µm.

### 4.1. Water-Based Sample Preparation and Analysis

To the sample containing distilled water with the PE standard, a drop of the surfactant Tween 20 (Sigma-Aldrich, Saint Louis, MO, USA) was added prior to plasma treatment. The samples were then exposed to cold plasma (CP) for time intervals ranging from 2.5 to 10 min. After plasma treatment, the samples were stained with Nile Red at a concentration of 0.3 mL of dye per 100 mL of sample. The sample staining procedure was performed based on modifications of the method proposed by Kang H. et al. [29]. Following staining, the samples were incubated in the dark for 30 min and subsequently observed under an optical microscope using red, yellow, or blue filters. Additionally, PE samples treated with CP in an aqueous matrix were examined under an SEM to observe potential structural changes in the particles. Samples containing distilled water with polyethylene particles are marked with the symbol—PE.time of contact of the sample with plasma, e.g., the control sample was marked: PE.0.

### 4.2. Preparation and Analysis of Treated Wastewater Samples

The subject of the study was treated wastewater originating from a municipal wastewater treatment plant, characterized by a total organic carbon (TOC) content of 10 mg/L. The corresponding chemical oxygen demand (COD) was approximately 25 mg O_2_/L, and the biochemical oxygen demand over five days (BOD_5_) was 12 mg O_2_/L. The pH of the wastewater was measured at 7.24, indicating a slightly neutral environment. The electrical conductivity (EC) was 730 µS/cm, which falls within the typical range for treated wastewater with a moderate content of dissolved mineral salts. ICP- OES analysis resulted in the following characteristics: phosphorus (P) 2 mg/L, sodium (Na) 30 mg/L, potassium 21 mg/L, calcium 70 mg/L, magnesium 16 m/L. Samples of treated wastewater (50 mL) containing the PE standard were subjected to cold plasma (CP) treatment in the same manner as the distilled water samples. However, preparation for microscopic observation after Nile Red staining was preceded by digestion of organic matter in the samples. Digestion was performed using 30% hydrogen peroxide (perhydrol) (Biomus sp. z o.o., Lublin, Poland) in a 1:1 ratio. The samples were incubated with H_2_O_2_ for 24 h at room temperature. The procedure for digesting organic matter in samples was performed based on information contained in the studies on the validation of microplastic sample preparation methods [30] and procedure optimization [31]. After digestion, the samples were centrifuged and rinsed three times with distilled water. They were then stained and observed under an optical microscope. Similarly, for samples containing treated sewage with polyethylene particles, the samples were marked with the symbol PE.W., corresponding to the time of contact of the sample with plasma, e.g., the control sample was marked: PE.W.0.

### 4.3. Preparation and Analysis of Polyethylene Samples Related to the Sorption of Pharmaceuticals on the Surface

Pharmaceuticals were added to the treated wastewater samples containing PE (Table 1). Each pharmaceutical was added individually at a concentration of 20 mg/L. The samples were incubated for 24 h on a shaker (180 rpm) at room temperature. Subsequently, the samples were subjected to plasma treatment for 5 min. After plasma exposure, the samples were pressure-filtered to eliminate solid particles. Additionally, the resulting permeate was passed through a syringe filter (Sigma-Aldrich, Saint Louis, MO, USA). The samples were then subjected to chromatographic analysis, the conditions of which are described in the publication [10]. The efficiency of pharmaceutical concentration reduction expressed in % was determined based on Equation (1):

(1) $$E\;=\;100\%\;-\;\left(\frac{C\;\times 100\%}{Cp}\right)$$

where E—efficiency of concentration reduction; Cp—initial pharmaceutical concentration; C—pharmaceutical concentration after the process.

To evaluate the sorption capacity of untreated polyethylene (PE) microparticles for selected pharmaceutical compounds, each drug was tested at its nominal initial concentration. Based on the recorded equilibrium concentrations (Ce) after 24 h of contact, the amount of drug adsorbed per gram of PE (q_e_) was calculated using the mass balance Equation (2):

(2) $$q_{e}\;=\;\;\frac{\left(Co\;-\;Ce\right)\;\times \;V}{m}$$

where Co—initial concentration (mg/L); Ce—equilibrium concentration (mg/L); V—volume of solution (L); m—mass of PE (g).

### 4.4. Energy Balance Calculations: Assumptions and Equations

An energy balance was prepared for the tested process using the Plasma TEC-X cold plasma generator (Table 5):

The energy balance was performed based on the calculation of the input energy, defined as the product of the total system power (P_t_) and treatment time. In the next step, the specific energy consumption (SEC) per 1% removal was calculated as the ratio of the input energy to the product of the sample volume and the percentage reduction in individual pharmaceuticals obtained after 5 min of cold plasma treatment. The estimation of energy costs was derived by multiplying the input energy by the average electricity price. Furthermore, the cost of achieving 1% reduction per 1 m^3^ of treated water was determined as the product of SEC and the average electricity price.

## Figures and Tables

**Table 1:** The dimensions of PE particles in individual samples were determined using the Fiji program (ImageJ 1.54p) in samples based on water.

Sample Name	Number of Objects in the Field of View	Area [µm^2^]	Width [µm]	Height [µm]	Perimeter [µm]
PE.0	1	1004.782	35.958	35.316	121.199
	2	547.951	26.326	26.326	88.535
	3	450.235	25.042	24.400	80.765
PE.2.5	1	1005.440	35.714	35.714	112.200
PE.5	1	1022.069	36.989	35.713	120.375
	2	682.464	31.887	29.336	100.186
PE.7.5	1	270.845	18.620	18,620	60.563
	2	965.065	35.313	34.671	115.146
PE.10	1	768.723	31.250	31.250	102.958
	2	685.749	28.699	29.974	97.173

**Table 2:** The dimensions of PE particles in individual samples were determined using the Fiji program (ImageJ 1.54p) in samples based on treated wastewater.

Sample Name	Number of Objects in the Field of View	Area [µm^2^]	Width [µm]	Height [µm]	Perimeter [µm]
PE.W.0	1	1184.848	43.073	39.273	151.286
PE.W.2.5	1	1532.530	46.232	43.232	146.846
	2	183.659	16.053	15.411	51.266
PE.W.5	1	400.396	23.274	22.627	79.710
PE.W.7.5	1	767.600	31.461	30.819	102.746
PE.W.10	1	1554.171	44.580	44.580	153.414

**Table 3:** Calculated values of specific energy consumption (SEC) and the corresponding unit treatment cost, based on an assumed electricity price of 0.15 EUR/kWh. These parameters provide an estimation of the energetic efficiency and economic feasibility of the cold plasma process.

Pollution	Reduction [%]	SEC [kWh/m^3^/1%]	Cost/m^3^/1% EUR
Sulfamethoxazole	99.99	49.45	7.42
Trimethoprim	98.11	50.38	7.56
Diclofenac	98.35	50.24	7.54
Carbamazepine	32.81	150.98	22.65
Caffeine	0	-	-

**Table 4:** Characteristics of the pharmaceutical substances used.

Substance	Therapeutic Group	Specification
Sulfamethoxazole (SMX)	Antibiotic	Crystalline powder, white or almost white. POL-AURA distributor (Zabrze, Poland)
Trimethoprim (TMP)	Antibiotic	White to pale yellow powder. POL-AURA distributor (Zabrze, Poland)
Diclofenac (DFC)	Non-steroidal anti-inflammatory drugs	Sodium salt, white powder. Sigma-Aldrich distributor (Saint Louis, MO, USA)
Carbamazepine (CBZ)	Anticonvulsant drug	Powder with a color ranging from white to off-white. POL-AURA distributor (Zabrze, Poland)
Caffeine (CAF)	Other	White powder. Biomus sp. z o.o. distributor (Lublin, Poland)

**Table 5:** Assumptions and input data used to perform the energy analysis of the conducted research.

Parameter	Input Value
Generator power	0.55 kW
Plasma nozzle power	0.415 kW
Air compressor	2 kW (at 4–8 bar, assuming continuous operation)
Total system power (P_t_)	2.965 kW
Operating time	5 min (0.0833 h)
Sample volume	50 mL (0.00005 m^3^)
Electricity price (E_p_)	(industrial, scenarios): 0.15 EUR/kWh (base); Sensitivity: 0.10–0.25 EUR/kWh
Initial pharmaceutical concentration	20 mg/L
Contaminant reduction (%) (R)	SMX 99.99%, TMP 98.11%, DFC 98.35%, CBZ 32.81% and CAF 0%

## Data Availability

Data is contained within the article.

## Abbreviations

The following abbreviations are used in this manuscript:

PE	Polyethylene
CP	Cold Plasma
SMX	Sulfamethoxazole
TMP	Trimethoprim
DFC	Diclofenac
CBZ	Carbamazepine
CAF	Caffeine

## References

[1] Dey T.K., Uddin M.E., Jamal M. (2021). Detection and removal of microplastics in wastewater: Evolution and impact. Environ. Sci. Pollut. Res..

[2] Sol D., Laca A., Laca A., Díaz M. (2021). Microplastics in wastewater and drinking water treatment plants: Occurrence and removal of microfibres. Appl. Sci..

[3] Bermúdez J.R., Swarzenski P.W. (2021). A microplastic size classification scheme aligned with universal plankton survey methods. MethodsX.

[4] Adhikari S., Kelkar V., Kumar R., Halden R.U. (2022). Methods and challenges in the detection of microplastics and nanoplastics: A mini-review. Polym. Int..

[5] Murugan P., Sivaperumal P., Balu S., Arya S., Atchudan R., Sundramoorthy A.K. (2023). Recent advances on the methods developed for the identification and detection of emerging contaminant microplastics: A review. RSC Adv..

[6] Klimasz M., Grobelak A. (2024). Accumulation of Spherical Microplastics in Earthworms Tissues-Mapping Using Raman Microscopy. Appl. Sci..

[7] Birania S., Attkan A.K., Kumar S., Kumar N., Singh V.K. (2022). Cold plasma in food processing and preservation: A review. J. Food Process Eng..

[8] Tabares F.L., Junkar I. (2021). Cold plasma systems and their application in surface treatments for medicine. Molecules.

[9] Mravlje J., Regvar M., Vogel-Mikuš K. (2021). Development of cold plasma technologies for surface decontamination of seed fungal pathogens: Present status and perspectives. J. Fungi.

[10] Wypart-Pawul A., Neczaj E., Grosser A., Grobelak A. (2024). Assessment of the effectiveness of atmospheric plasma on the removal of selected pharmaceuticals from water. Desalination Water Treat..

[11] Gururani P., Bhatnagar P., Bisht B., Kumar V., Joshi N.C., Tomar M.S., Pathak B. (2021). Cold plasma technology: Advanced and sustainable approach for wastewater treatment. Environ. Sci. Pollut. Res..

[12] Caban R., Gnatowski A. (2024). Analysis of the Impact of Waste Fly Ash on Changes in the Structure and Thermal Properties of the Produced Recycled Materials Based on Polyethylene. Materials.

[13] Swain R., Nagamani R., Panda S. (2015). Formulation, in vitro Characterization and Stability Studies of Fast Dispersing Tablets of Diclofenac Sodium. J. Appl. Pharm. Sci..

[14] Hussain Z., Yousif E., Ahmed A., Altaie A. (2014). Synthesis and characterization of Schiff’s bases of sulfamethoxazole. Org. Med. Chem. Lett..

[15] ElShaer A., Hanson P., Worthington T., Lambert P., Mohammed A.R. (2012). Preparation and Characterization of Amino Acids-Based Trimethoprim Salts. Pharmaceutics.

[16] Turicek J., Ratts N., Kaltchev M., Masoud N. (2021). Surface treatment of ultra-high molecular weight polyethylene (UHMWPE) by cold atmospheric plasma (CAP) for biocompatibility enhancement. Appl. Sci..

[17] Wang C.Y., Schön M., Horn T., Facklam M., Dahlmann R., Hopmann C., He G.J. (2022). Usage of atmosphere pressure plasma for rapid polyethylene functionalisation exhibiting only minor ageing. Eur. Polym. J..

[18] Chen C., Taghavi N., Baroutian S. (2024). Effect of cold plasma pretreatment on biodegradation of high-density polyethylene (HDPE) and polystyrene (PS). J. Mater. Cycles Waste Manag..

[19] Chytrosz-Wrobel P., Golda-Cepa M., Stodolak-Zych E., Rysz J., Kotarba A. (2023). Effect of oxygen plasma-treatment on surface functional groups, wettability, and nanotopography features of medically relevant polymers with various crystallinities. Appl. Surf. Sci. Adv..

[20] Yao L., King J., Wu D., Chuang S.S.C., Peng Z. (2021). Non-thermal plasma-assisted hydrogenolysis of polyethylene to light hydrocarbons. Catal. Commun..

[21] Guo X., Chen C., Wang J. (2019). Sorption of sulfamethoxazole onto six types of microplastics. Chemosphere.

[22] Razanajatovo R.M., Ding J., Zhang S., Jiang H., Zou H. (2018). Sorption and desorption of selected pharmaceuticals by polyethylene microplastics. Mar. Pollut. Bull..

[23] McDougall L., Thomson L., Brand S., Wagstaff A., Lawton L.A., Petrie B. (2022). Adsorption of a diverse range of pharmaceuticals to polyethylene microplastics in wastewater and their desorption in environmental matrices. Sci. Total Environ..

[24] Kanteraki A., Isari E.A., Grilla E., Giotis K., Kalavrouziotis I., Svarnas P. (2024). Pharmaceutically Active Compound (PhAC) Degradation by Means of Cold Plasma Jet Treatment. Plasma.

[25] Guo H., Wang Y., Wang J., Tang S., Wang T. (2025). Review on application of non-thermal plasma for disinfection: Direct plasma and indirect plasma-activated water. Chin. Chem. Lett..

[26] Rayaroth M.P., Aubry O., Rabat H., Marilleau E., Gru Y., Hong D., Vaudin P., Brault P. (2025). Non-Thermal Plasma Oxidation Processes for the Removal of Pharmaceuticals in Water: Diclofenac and Diclofenac/Carbamazepine Mixture. Plasma Process. Polym..

[27] Rayaroth M.P., Aubry O., Rabat H., Marilleau E., Gru Y., Hong D., Brault P. (2024). Degradation and transformation of carbamazepine in aqueous medium under non-thermal plasma oxidation process. Chemosphere.

[28] Yin Y., Xu H., Zhu Y., Zhuang J., Ma R., Cui D., Jiao Z. (2023). Recent Progress in Applications of Atmospheric Pressure Plasma for Water Organic Contaminants’ Degradation. Appl. Sci..

[29] Kang H., Park S., Lee B., Ahn J., Kim S. (2020). Modification of a nile red staining method for microplastics analysis: A nile red plate method. Water.

[30] Al-Azzawi M.S.M., Knoop O., Drewes J.E. (2022). Validation of sample preparation methods for small microplastics (≤10 µm) in wastewater effluents. Chem. Eng. J..

[31] Sturm M.T., Myers E., Korzin A., Polierer S., Schober D., Schuhen K. (2023). Fast Forward: Optimized Sample Preparation and Fluorescent Staining for Microplastic Detection. Microplastics.

